# Contribution of preoperative gut microbiota in postoperative neurocognitive dysfunction in elderly patients undergoing orthopedic surgery

**DOI:** 10.3389/fnagi.2023.1108205

**Published:** 2023-02-17

**Authors:** Jiangjiang Bi, Yifan Xu, Shiyong Li, Gaofeng Zhan, Dongyu Hua, Juan Tan, Xiaohui Chi, Hongbing Xiang, Fengjing Guo, Ailin Luo

**Affiliations:** ^1^Department of Anesthesiology, Hubei Key Laboratory of Geriatric Anesthesia and Perioperative Brain Health, and Wuhan Clinical Research Center for Geriatric Anesthesia, Tongji Hospital, Tongji Medical College, Huazhong University of Science and Technology, Wuhan, China; ^2^Department of Orthopedics, Tongji Hospital, Tongji Medical College, Huazhong University of Science and Technology, Wuhan, China

**Keywords:** gut microbiota, metabolites, orthopedic surgery, elderly patients, postoperative neurocognitive dysfunction

## Abstract

**Objective:**

To investigate the role of gut microbiota and metabolites in POCD in elderly orthopedic patients, and screen the preoperative diagnostic indicators of gut microbiota in elderly POCD.

**Method:**

40 elderly patients undergoing orthopedic surgery were enrolled and divided into Control group and POCD group following neuropsychological assessments. Gut microbiota was determined by 16S rRNA MiSeq sequencing, and metabolomics of GC–MS and LC–MS was used to screen the differential metabolites. We then analyzed the pathways enriched by metabolites.

**Result:**

There was no difference in alpha or beta diversity between Control group and POCD group. There were significant differences in 39 ASV and 20 genera bacterium in the relative abundance. Significant diagnostic efficiency analyzed by the ROC curves were found in 6 genera bacterium. Differential metabolites in the two groups including acetic acid, arachidic acid, pyrophosphate etc. were screened out and enriched to certain metabolic pathways which impacted the cognition function profoundly.

**Conclusion:**

Gut microbiota disorders exist preoperatively in the elderly POCD patients, by which there could be a chance to predict the susceptible population.

**Clinical Trial Registration:**

[http://www.chictr.org.cn/edit.aspx?pid=133843&htm=4], identifier [ChiCTR2100051162].

## 1. Introduction

Studies have found that the elderly are more prone to suffer from postoperative neurocognitive dysfunction (POCD; Moller et al., 1998), and our country is irreversibly entering an ageing society. It is predicted that by 2050, approximately 50% of patients undergoing general anaesthetic surgery will be aged 65 years or older, and POCD seriously affects patients’ postoperative quality of life, prolongs hospital stays, and even increases the morbidity and mortality rate, putting enormous pressure on families and society (Skvarc et al., 2018). Therefore, it is urgent to study the pathogenesis of POCD in the elderly and explore its treatment.

Postoperative cognitive dysfunction (POCD) which has received widespread attention in recent years, refers to the development of central nervous system complications in the elderly after surgery, manifesting as confusion, anxiety, personality changes and memory impairment (Le et al., 2014; Tian et al., 2017). Some studies suggest that the incidence of POCD in elderly patients undergoing elective surgery under general anesthesia is two to ten times higher than in younger patients. Thus, advanced age is an important risk factor in its pathogenesis.

POCD is known to be similar to AD in terms of pathogenesis, clinical presentation, and morphopathological changes within the central nervous system (Evered et al., 2016; Fang et al., 2019). However, the diagnosis of POCD relies on the anesthesiologist’s own experience and cognitive scales, and its sensitivity for early diagnosis of POCD is poor. Therefore, the development of objective diagnostic indicators for POCD is an important issue that needs to be addressed in clinical anesthesia research.

Naseer et al. (2014) showed that the composition and numbers of gut microbiota in AD patients were significantly different from those of healthy controls. In a clinical study of Parkinson’s, Mulak and Bonaz (2015) concluded that the alpha and beta distribution of gut microbiota was different from that of their control group, suggesting the composition and function of the gut microbiota in Parkinson’s patients is different from that of healthy controls. Zhan et al. (2018) found that the alpha and beta distributions of the gut microbiota of SAMP8 mice were significantly different from those of control mice. In addition, they found a significant improvements in behavioral scores of cognitive function in this group of mice after gastrointestinal colonisation of pseudo-sterile mice with SAMR1 mouse fecal bacteria. These results all suggest an association between cognitive dysfunction and gut microbiota disorders. Even so, the relationships between gut microbiota and POCD in human have not been studied extensively. In this study, we investigated the role of gut microbiota and metabolites in POCD in elderly orthopedic patients, and tried to seek for the preoperative diagnostic indicators of gut microbiota in elderly POCD patients.

## 2. Materials and methods

### 2.1. Clinical research design

This was a prospective case–control study to collect fecal samples from elderly patients undergoing orthopedic surgery, to compare changes in gut microbiota and metabolites between POCD and control patients. The study was approved by the Medical Ethics Committee of Tongji Hospital affiliated to Tongji Medical College, Huazhong University of Science and Technology. All patients in the research signed informed consent before sample collection.

Inclusion criteria: Patients undergoing elective internal fixation of lower limb fractures, knee replacement or hip replacement by general anesthesia, American Society of Anesthesiologists (ASA) classification I – II, age 66–84 years old, and conscious in perioperative period were enrolled.

Exclusion Criteria: Patients suffered from one of following terms were excluded: central nervous system diseases or psychological disorders, long-term use of sedatives or antidepressants within the last year, Parkinson’s disease, severe hearing or visual impairment, drug dependence, alcoholism, inability to communicate with a physician.

The PASS 15 software was applied to calculate the sample size. The study is a diagnostic test and test for One Receiver Operating Characteristic (ROC) curve was applied. Existing studies show that the incidence of POCD in the elderly is about 40%, the sample size of the positive group was set at 40%, the AUC was 0.8, the type I error α was 0.05, the type II error β was 0.1 and the drop out rate was 20%, resulting in a positive sample size of 16 cases and a negative sample size of 24 cases for the study. The total sample size for this study was 40 cases.

### 2.2. General information collection

The necessarily general information and medical history were collected with permission of each patient before surgery. Information about vital signs during the operation period, postoperative pain score and antibiotics treatment were also collected.

### 2.3. Neuropsychological assessment

Neuropsychological assessments were performed to each patient 1 day before surgery, 1 day after surgery, 3 days after surgery and 28 days after surgery. The cognitive function of patients were assessed using the Mini-mental state examination (MMSE), Montreal cognitive assessment-basic (MoCA-B) and the Brief mental status assessment (Mini-Cog) and all the participant were divided into POCD and Control groups based on the assessment results. All the assessments above were performed by the same anesthetist. Adapted questions of MoCA-B facilitate the detection of mild cognitive impairment in subjects who are illiterate or possess a low education level. MoCA-B assesses executive function, language, orientation, calculation, etc., and is available for clinical research from official website.^1^

### 2.4. DNA extraction and amplification of fecal samples

Fecal samples (about weight 2 g) were collected 1 day before surgery, suspended in fecal storage solution (Langfu Biotechnology Corporation, Shanghai, China) and then snap frozen and stored at −80°C. Bacterial DNA was isolated from the fecal samples using a MagPure Soil DNA LQ Kit (Magen, Guangdong, China) following the manufacturer’s instructions. DNA concentration and integrity were measured by a NanoDrop 2000 spectrophotometer (Thermo Fisher Scientific, Waltham, MA, United States) and agarose gel electrophoresis, respectively. PCR amplification of the V3-V4 hypervariable regions of the bacterial 16S rRNA gene was carried out in a 25 μl reaction using universal primer pairs (343F: 5′TACGGRAGGCAGCAG-3′; 798R: 5′- AGGGTATCTAATCCT-3′). The reverse primer contained a sample barcode and both primers were connected with an Illumina sequencing adapter.

### 2.5. 16S rRNA gene sequencing

The Amplicon quality was visualized using gel electrophoresis. The PCR products were purified with Agencourt AMPure XP beads (Beckman Coulter Co., USA) and quantified using Qubit dsDNA assay kit. The concentrations were then adjusted for sequencing which was performed on an Illumina NovaSeq6000with two paired-end read cycles of 250 bases each (Illumina Inc., San Diego, CA; OE Biotech Company, Shanghai, China).

Raw sequencing data were in FASTQ format. Paired-end reads were then preprocessed using cut adapt software to detect and cut off the adapter. After trimming, paired-end reads were filtering low quality sequences, denoised, merged and detect and cut off the chimera reads using DADA2 (Callahan et al., 2016) with the default parameters of QIIME2 (Bolyen et al., 2019). At last, the software output the representative reads and the ASV abundance table.

### 2.6. Untargeted metabolomics analysis GC–MS and LC–MS

#### 2.6.1. GC–MS

The samples were analyzed on an Agilent 7890B gas chromatography system coupled to an Agilent 5977AMSD system (Agilent TechnologiesInc., CA, United States). ADB-5MS fused silica capillary column (30 m × 0.25 mm × 0.25 μm, Agilent J & W Scientific, Folsom, CA, United States) was utilized to separate the derivatives. Helium (>99.999%) was used as the carrier gas at a constant flow rate of 1 ml/min through the column.

#### 2.6.2. LC–MS

A Dionex Ultimate 3,000 RS UHPLC fitted with Q-Exactive plus quadrupole-Orbitrap mass spectrometer equipped with heated electrospray ionization (ESI) source (Thermo Fisher Scientific, Waltham, MA, United States) was used to analyze the metabolic profiling in both ESI positive and ESI negative ion modes. An ACQUITY UPLC HSS T3 column (1.8 μm, 2.1 × 100 mm) were employed in both positive and negative modes.

## 3. Statistical analysis and bioinformatic analysis

Statistical analysis was performed using SPSS 17.0 (SPSS Inc., Armonk, New York, United States). Values presented were expressed as mean ± standard error of the mean (S.E.M.), comparisons between groups were performed using one-way analysis of variance (ANOVA) followed by *post hoc* Tukey tests or Fisher’s exact tests. Normal distribution data were analyzed using one-way ANOVA, whereas non-normal distribution data were analyzed using Fisher’s exact test. Comparisons of numbers of variables in the general information between groups were performed by Chi-square test and Fisher’s exact test. Diagnostic cut-offs, AUC, sensitivity and specificity were determined by ROC curve analysis. *p*-value <0.05 is considered to be a significant difference.

The representative read of each ASV was selected using QIIME 2 package. All representative reads were annotated and blasted against Silva database Version 138 (16 s rDNA) using q2-feature-classifier with the default parameters. The microbial diversity in fecal samples was estimated using the alpha diversity that include Chao1 index, Shannon index and ACE index. The Unifrac distance matrix performed by QIIME2 software was used for unweighted Unifrac Principal coordinates analysis (PCoA) and phylogenetic tree construction. The 16S rRNA gene amplicon sequencing and analysis were conducted by OE Biotech Co., Ltd. (Shanghai, China).

Multiple statistical analyses were performed using principal component analysis (PCA), partial least squares analysis (PLS-DA) and orthogonal partial least squares analysis (OPLS-DA) to find the differential metabolites between groups. Student’s T test and a Fold change analysis were used to compare metabolites between the two groups. A combination of multidimensional and unidimensional analyses was used to screen for differential metabolites. Metabolic pathways enrichment analysis of differential metabolites were performed based on the Kyoto Encyclopedia of Genes and Genomes (KEGG) database. The screening criteria was VI*p* value >1 for the first principal component of the OPLS-DA model and value of *p* <0.05 for the *T*-test.

## 4. Results

### 4.1. General information and perioperative clinical data

General information and medical history were listed in Table 1. Neuropsychological assessment outcomes including all time points were listed in Table 2. Postoperative pain numeric rating scales (NRS) were listed in Table 3.

**Table 1:** General informations and medical history of the participants.

Items	Control group (*n* = 16)	POCD group (*n* = 16)	Value of *p*
Age (years)	71.38 ± 0.94	71.81 ± 1.04	0.762
66 ~ 70	7	6	0.986
71 ~ 75	7	8	
76 ~ 80	1	1	
81 ~ 85	1	1	
Gender (male/female)	10/6	5/11	0.077
Type of surgery			0.865
Knee replacement	8	8	
Hip replacement	5	6	
Internal fixation for fracture of lower limb	3	2	
Hypertension (number)	10	9	0.719
Diabetes (number)	5	1	0.070
HLP (number)	1	1	1.000
Stroke (number)	1	4	0.333
ECG abnormal (number)*	6	3	0.433
Glu (mmol/L)	6.18 ± 0.63	5.83 ± 0.50	0.679
BUN (mmol/L)	5.87 ± 0.51	6.07 ± 0.39	0.681
INR	0.99 ± 0.02	1.01 ± 0.02	0.615
LVEF (%)	64.79 ± 0.85	66.44 ± 0.88	0.286

* ECG abnormal: ST, segment ischemia performance; HLP, hyperlipidaemia; CHD, chronic heart disease; Glu, Blood glucose; BUN, blood urea nitrogen; INR, international normalized ratio; LVEF, left ventricular ejection fraction.

**Table 2:** Differences of neuropsychological assessment outcomes between the two groups.

Items	Postoperative time (day)	Control group (*n* = 16)	POCD group (*n* = 16)	value of *p*
MoCA-B	1st	23.13 ± 0.64	12.31 ± 1.21	< 0.0001*
	3rd	23.50 ± 0.93	13.94 ± 1.27	< 0.0001*
	28th	24.22 ± 0.53	13.23 ± 1.12	< 0.0001*
Mini-Cog	1st	3.31 ± 0.48	0.81 ± 0.23	0.0006*
	3rd	3.87 ± 0.27	1.12 ± 0.22	< 0.0001*
	28th	4.13 ± 0.27	1.94 ± 0.28	< 0.0001*
MMSE	28th	28.44 ± 0.58	21.63 ± 1.22	0.0003*

^*^p < 0.05.

**Table 3:** Differences of postoperative pain NRS between the two groups.

Postoperative time	Control group (*n* = 16)	POCD group (*n* = 16)	Value of *p*
Recovery	3.38 ± 0.60	2.44 ± 0.39	0.140
1st day	3.00 ± 0.35	3.06 ± 0.44	0.905
3rd day	2.69 ± 0.44	2.94 ± 0.37	0.705
7th day	1.56 ± 0.35	1.63 ± 0.33	0.894

### 4.2. Microbiological composition in POCD patients

The total data volume of raw reads after sequencing ranged from 78,268 to 81,928, clean tags after quality control ranged from 59,473 to 73,165, clean tags after removing chimeras to obtain valid tags ranged from 42,922 to 70,784. The number of ASVs in each sample ranged from 103 to 533. Comparisons between the two groups using Wilcoxon rank sum test, significant differences were observed in 39 ASVs and 20 genus.

**Figure1:**
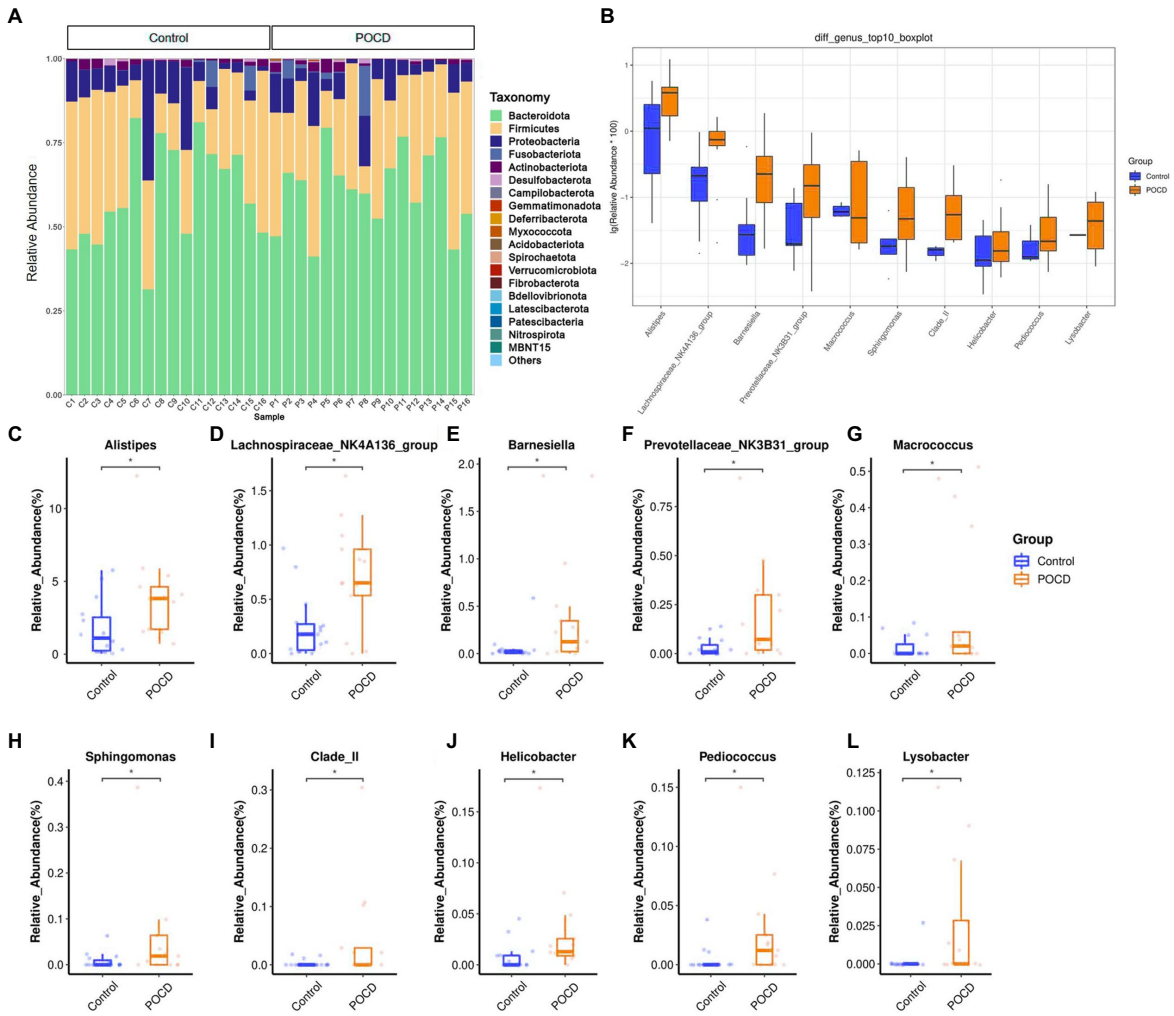
Comparisons of relative abundance of gut microbiota. **(A)** Histogram results for the top 20 in order of relative abundance at phylum level, which shows Bacteroidota, Firmicutes, Proteobacteria etc. contribute the main components of gut microbiota, **(B)** Comparisons between the two groups using Wilcoxon rank sum test, significant differences in relative abundance were observed in 20 genus (*p < 0.05), and **(C**–**L)** Relative abundance of differential genera bacterium, analyzed by two-tailed Wilcoxon rank-sum test, data represent the means ± SEM, *n* = 16 in each group, *p < 0.05 compared with Control group.

The statistical result showed the top relative abundance at 6 levels, including *Bacteroidota, Firmicutes, Proteobacteria, Actinobacteriota, Fusobacteria* were the top abundance at the phylum level (Figure 1A). It showed significant differences between the two groups using Wilcoxon rank sum test in the relative abundance at the genus level, including *Alistipes* (*p* = 0.008), *Helicobacte*r (*p* = 0.007), *Lysobacter* (*p* = 0.011), *Barnesiella* (*p* = 0.017), *Macrococcu*s (*p* = 0.049), etc. (Figure 1B). Relative abundance of top 10 differential genera bacterium were listed in Figures 1C–L.

**Figure 2:**
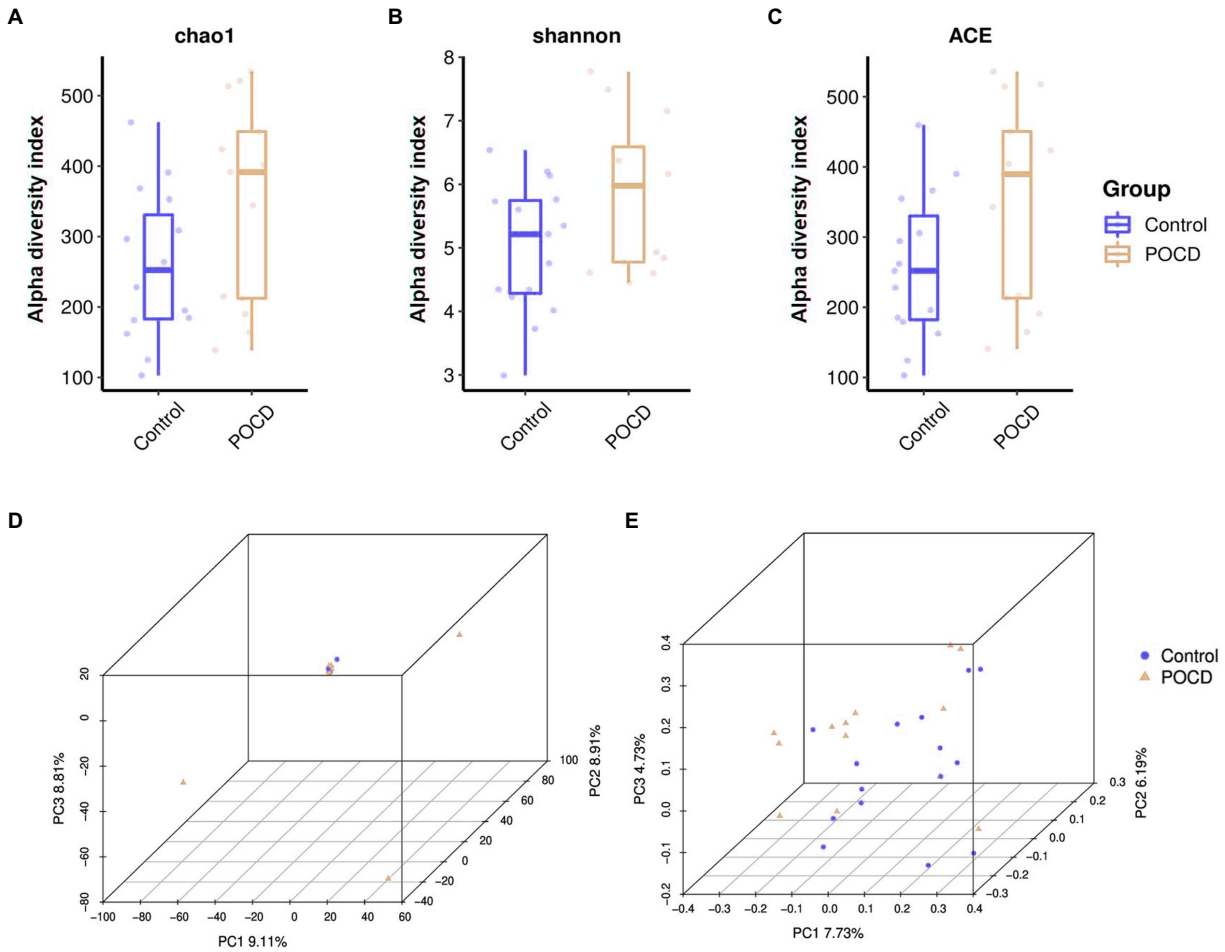
Alpha diversity and beta diversity in gut microbiota between the two groups. **(A–C)** There was no significant difference of alpha diversity as measured by the Chao1 diversity index (*p* = 0.098). The Shannon index (*p* = 0.065) and ACE index (*p* = 0.110) indicated species diversity and evenness, did not show significant differences. **(D,E)** There was no difference between the two groups measured by PCA and PCoA.

There was no significant difference of alpha diversity as measured by the Chao1 diversity index (*p* = 0.098). The Shannon index (*p* = 0.065) and ACE index (*p* = 0.110) indicated species diversity and evenness, did not show significant differences either (Figures 2A–C). There was no difference between the two groups measured by PCA and PCoA which indicated beta diversity based on the Bray-Curtis distance algorithm (Figures 2D,E).

### 4.3. Evaluation of gut bacteria for diagnosis of POCD using ROC curve analysis

**Figure 3:**
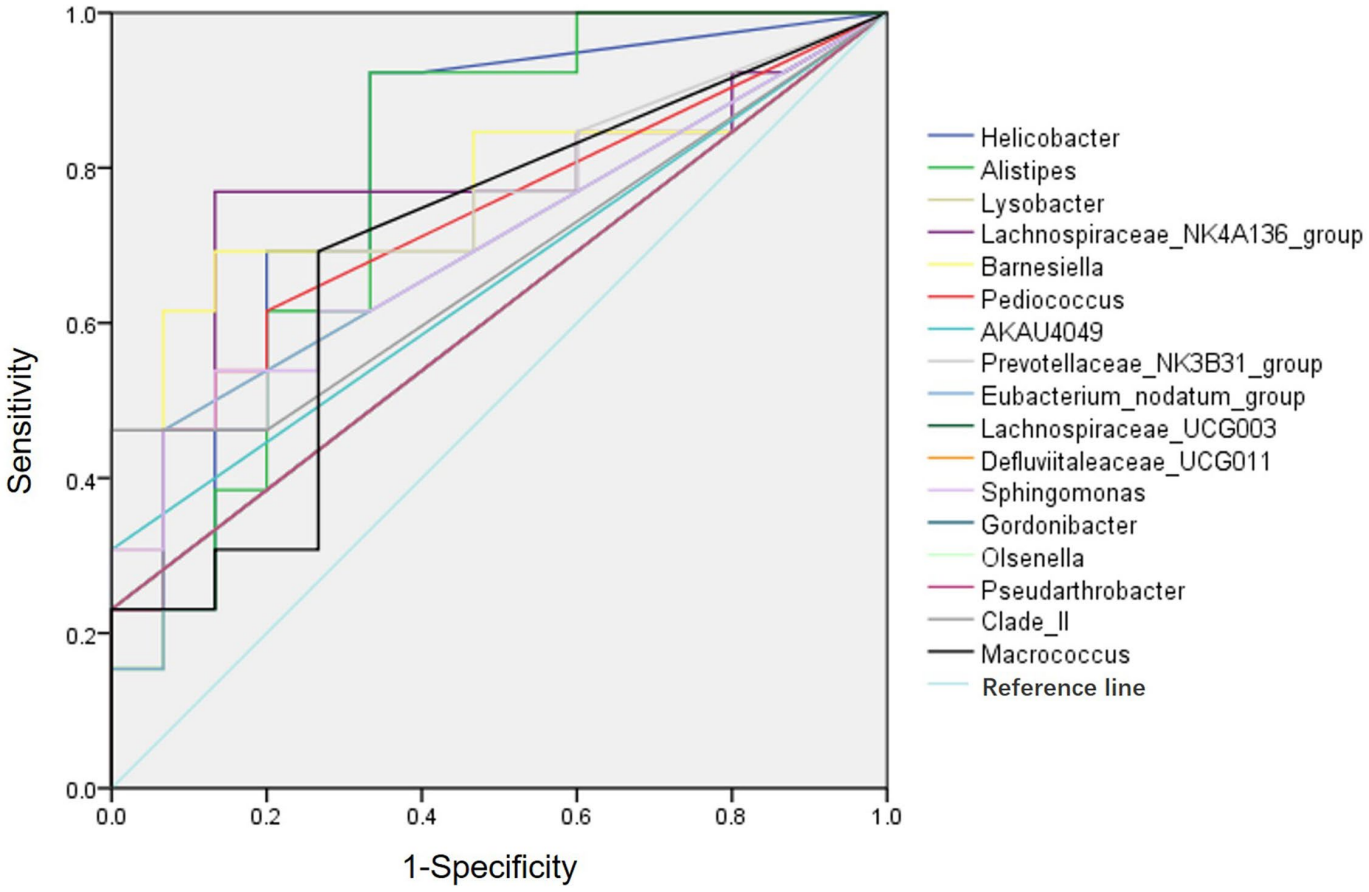
ROC curves of the gut genus bacterium relative abundance for the diagnosis of POCD. Vertical coordinate indicated the sensitivity of diagnosis, horizontal coordinate indicated the 1-specificity of diagnosis, AUC > 0.5 indicated a diagnosis efficiency of the gut bacterium.

ROC curve analysis was performed to evaluate the diagnostic efficiency of the 20 differential genera gut bacteria in POCD (Figure 3). The diagnostic efficiency of genera *Helicobacter, Alistipes, Barnesiella, Lachnospiraceae_NK4A136_group, Prevotellaceae_NK3B31_group*, and *Pediococcus* were significantly higher in POCD group than those in Control group. The area under curve (AUC), *p* value, sensitivity, specificity of gut bacteria for the diagnosis of POCD are listed in Table 4.

**Table 4:** ROC curve analysis of evaluation of gut bacteria for diagnosis of POCD.

Differential genus	AUC	value of *p*	Sensitivity	Specificity
Helicobacter	0.792	0.009*	0.923	0.667
Alistipes	0.779	0.012*	0.923	0.667
Lachnospiraceae_NK4A136_group	0.764	0.018*	0.769	0.867
Barnesiella	0.749	0.025*	0.692	0.867
Prevotellaceae_NK3B31_group	0.738	0.032*	0.462	1.000
Pediococcus	0.728	0.040*	0.615	0.800
Lysobacter	0.703	0.069	0.462	0.933
AKAU4049	0.654	0.167	0.308	1.000
Veillonella	0.333	0.134	0.000	0.667
Eubacterium_nodatum_group	0.692	0.084	0.462	0.933
Lachnospiraceae_UCG-003	0.615	0.300	0.231	1.000
Defluviitaleaceae_UCG-011	0.615	0.300	0.231	1.000
Sphingomonas	0.703	0.069	0.538	0.867
Family_XIII_UCG-001	0.367	0.231	0.000	0.733
GCA-900066575	0.697	0.076	0.615	0.800
Gordonibacter	0.615	0.300	0.231	1.000
Olsenella	0.615	0.300	0.231	1.000
Pseudarthrobacter	0.615	0.300	0.231	1.000
Clade_II	0.677	0.112	0.462	1.000
Macrococcus	0.692	0.084	0.692	0.733

*p < 0.05.

### 4.4. Differential metabolites and related metabolic pathways

**Figure 4:**
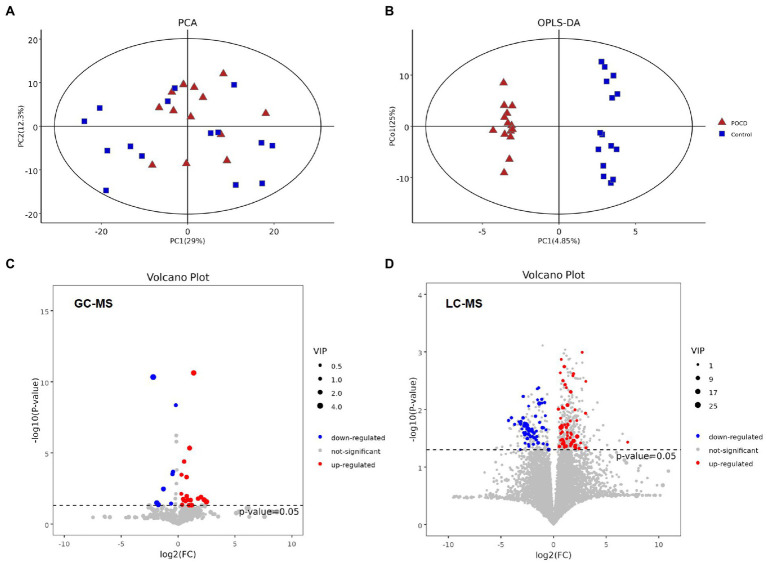
**(A,B)** It showed significant differences between two groups at the metabolic profiling level both in PCA and OPLS-DA (*R*^2^ = 0.961, *Q*^2^ = −0.268), **(C,D)** Differential metabolites between the two groups performed by T-test, and visualization by their value of ps and Fold change values using volcano plots. The red dots represent metabolites that were significantly upregulated in the POCD group, the blue dots represent metabolites that were significantly downregulated and the grey dots represent metabolites that were not significant.

**Figure 5:**
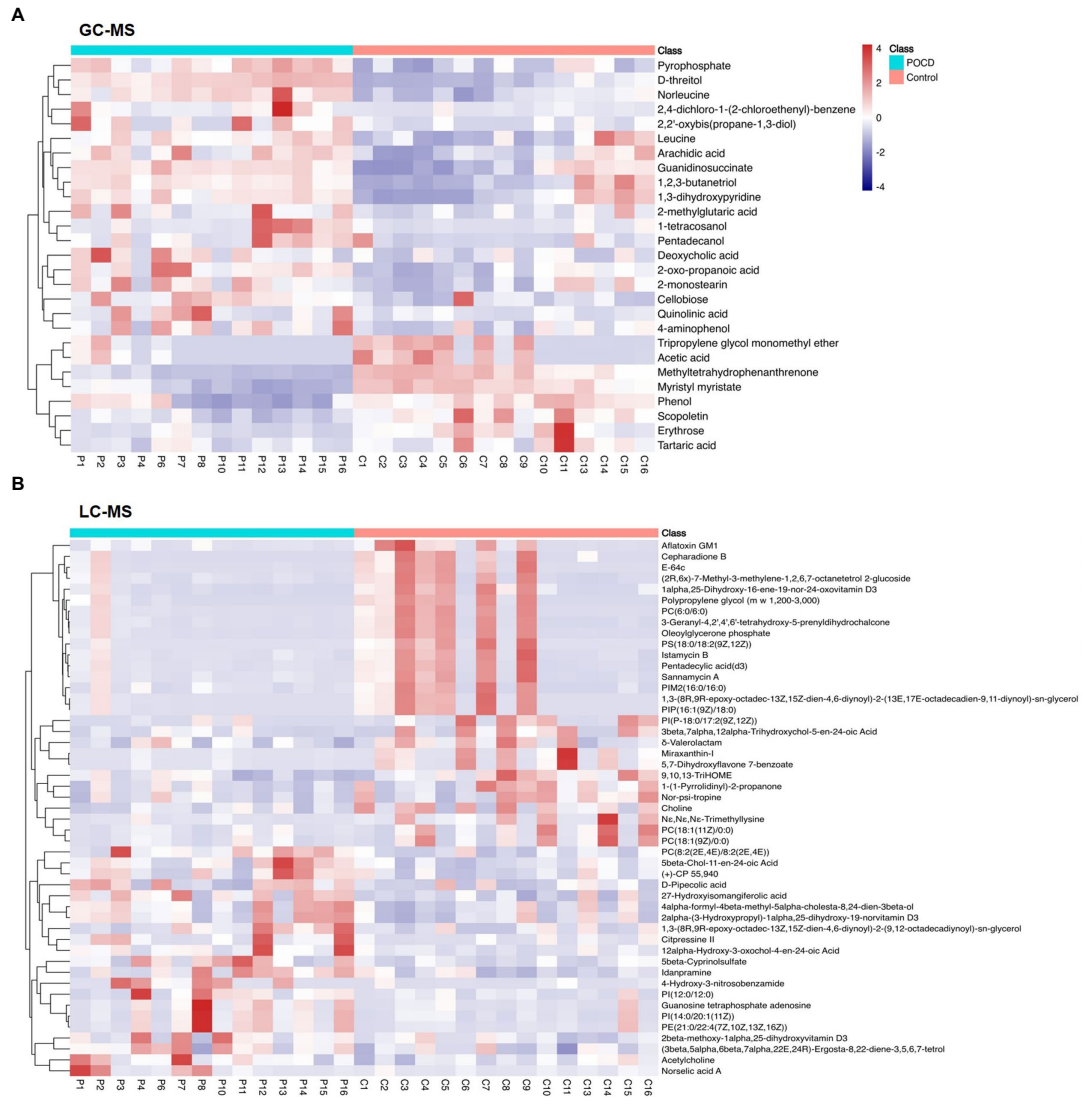
Heat map of differential metabolites. We performed hierarchical clustering of all significantly differential metabolites expressions, including 27 metabolites in GC–MS **(A)**, and 125 metabolites in LC–MS **(B)**. The horizontal coordinates indicate sample names and the vertical coordinates indicate differential metabolites. The colour ranges from blue to red, a redder colour indicates a higher abundance of expression of the differential metabolite.

Untargeted metabolomics analysis including GC–MS and LC–MS platforms were performed, and significant differences were found between two groups at the metabolic profiling level using PCA, PLS-DA and OPLS-DA (*R*^2^ = 0.961, *Q*^2^ = −0.268; Figures 4A,B). A total of 27 differential metabolites were screened out by *T*-test and visualized by volcano plots (Figures 4C,D) and Hierarchical Clustering in the GC–MS platform, including Acetic acid, Leucine, Pyrophosphate, etc. (Figure 5). While 125 differential metabolites were screened out in the LC–MS platform. The differential metabolites were listed in Supplementary materials Data sheet 1 and 2.

**Figure 6:**
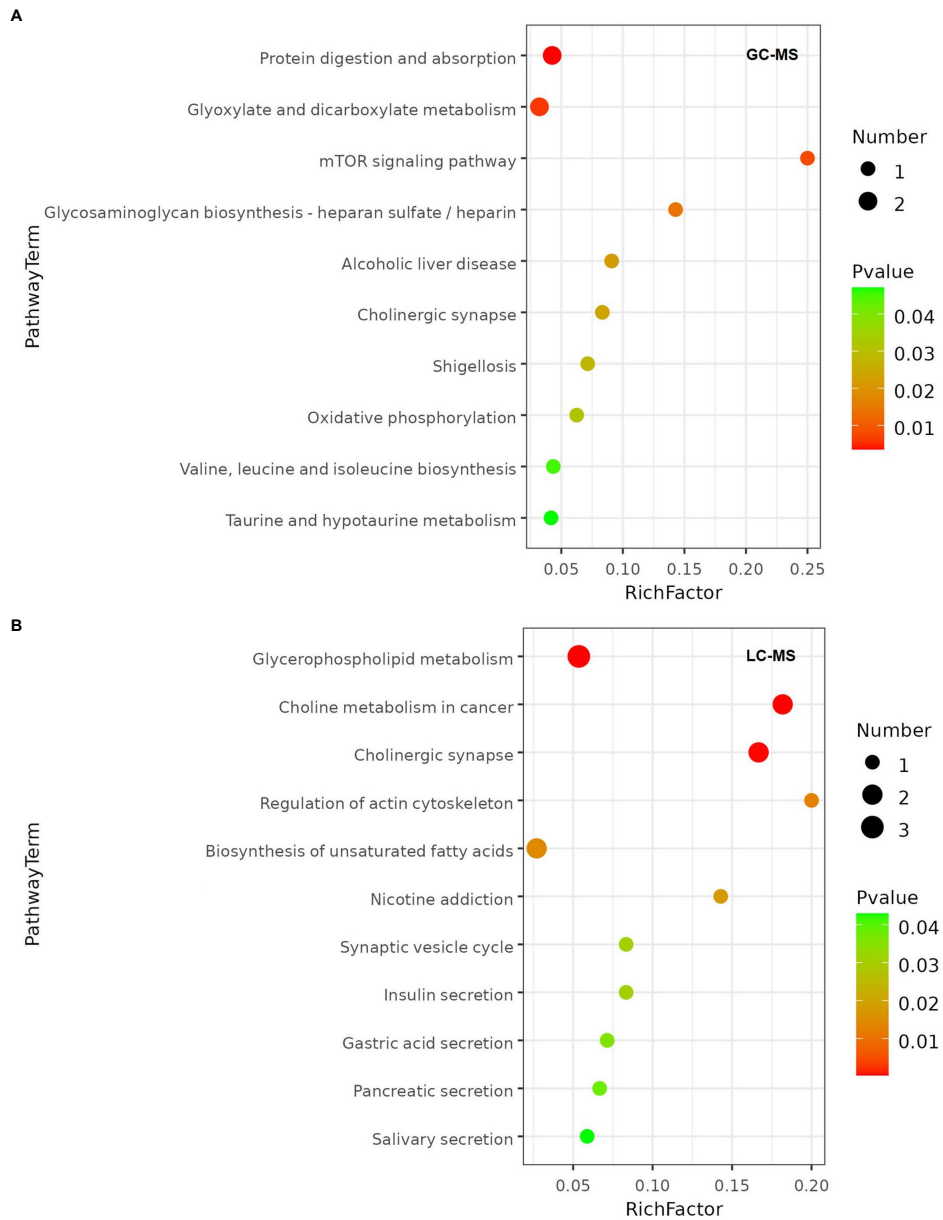
Metabolic pathway enrichment bubble chart. The value of *p* of the metabolic pathway is the significance of the enrichment of the metabolic pathway, and the significant enrichment pathway was selected for bubble plotting. The vertical coordinate is the name of the metabolic pathway; the horizontal coordinate is the enrichment factor (Rich factor = number of significantly differential metabolites/total number of metabolites in the pathway), the larger the Rich factor, the greater the enrichment; the colour from green to red indicates that the value of *p* decreases; the larger the bubble, the greater the number of metabolites enriched to that pathway. **(A)** Metabolic pathway enrichment of GC-MS platform. **(B)** Metabolic pathway enrichment of LC-MS platform.

Metabolic pathways highly enriched by the differential metabolites were then found out using Hypergeometric test based on the KEGG database. The pathways with top richness metabolites in the GC–MS platform were: Protein digestion and absorption, Glyoxylate and dicarboxylate metabolism, mTOR signaling pathway, Glycosaminoglycan biosynthesis-heparan sulfate/heparin, Alcoholic liver disease, Cholinergic synapse, Shigellosis, Oxidative phosphorylation, Valine, leucine and isoleucine biosynthesis, Taurine and hypotaurine metabolism (*p* < 0.05; Figure 6A). And the top enriched metabolic pathways in LC–MS platform were: Glycerophospholipid metabolism, Choline metabolism in cancer, Cholinergic synapse, Regulation of actin cytoskeleton, Biosynthesis of unsaturated fatty acids, Nicotine addiction, Synaptic vesicle cycle, Insulin secretion, Gastric acid secretion, Pancreatic secretion, Salivary secretion (*p* < 0.05; Figure 6B).

### 4.5. Correlation between microbiome and metabolites

**Figure 7:**
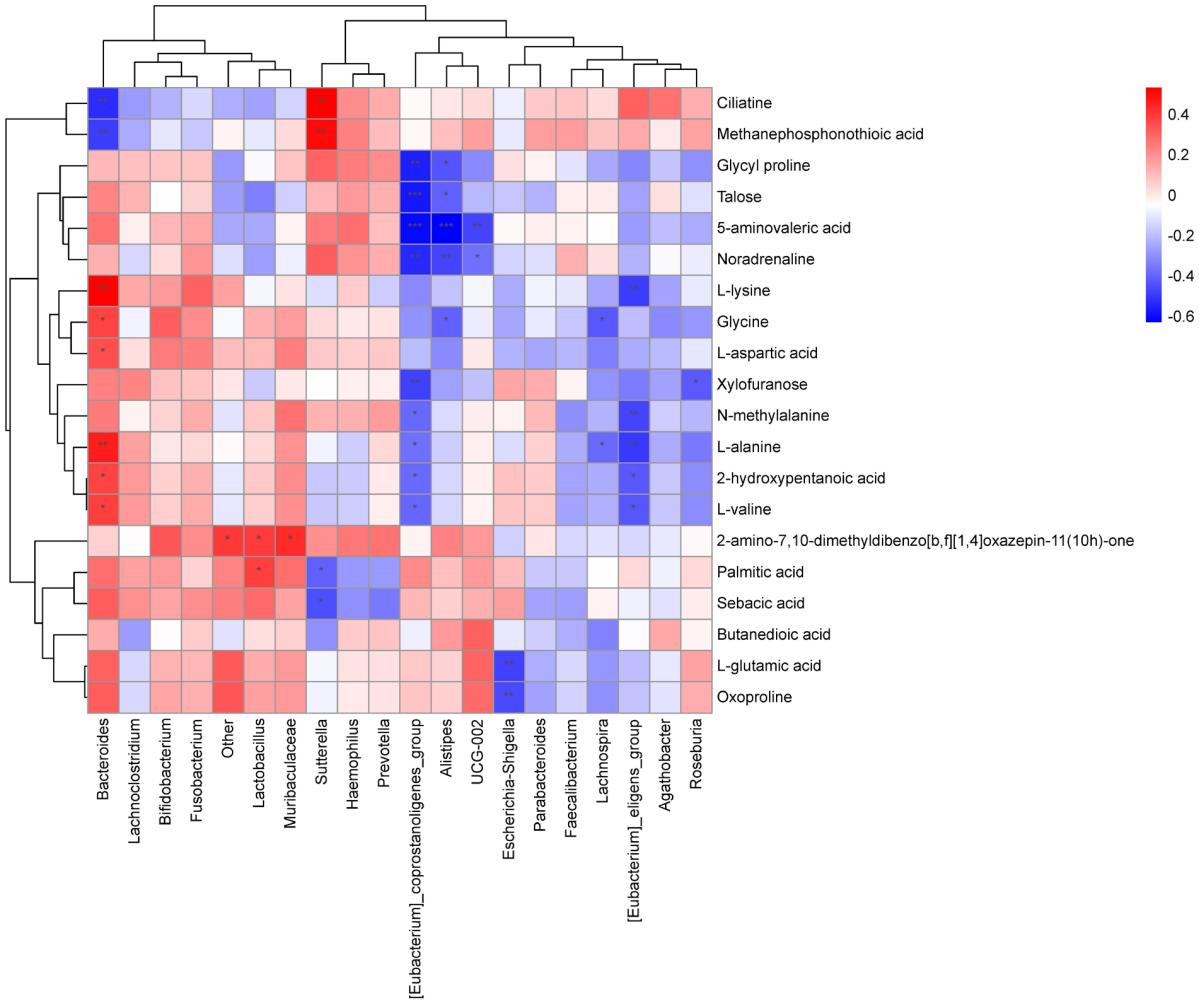
Heat map of correlation between microbiome and metabolites. Each column is for a different species and each row corresponds to a metabolite. The graph shows positive correlations in orange and negative correlations in blue, with darker colours representing greater correlations and colours closer to white representing correlations closer to zero. A *** in the graph represents a correlation *p* value less than 0.001, a ** in the graph represents a correlation p value less than 0.01 and a * in the graph represents a correlation *p* value less than 0.05.

Spearman correlation was applied to analyze the correlation between gut microbiota and metabolites and the results indicated significant correlations between them. Bacteroides was significantly negative correlated to Methanephosphonothioic acid (*p* = 0.005) and Ciliatine (*p* = 0.002), and positive correlated to L-lysine (*p* = 0.025) and L-alanine (*p* = 0.040). While Sutterella was positive correlated to Methanephosphonothioic acid (*p* = 0.003) and Ciliatine (*p* = 0.002). [Eubacterium]_coprostanoligenes_group was negative correlated to 5-aminovaleric acid (*p* = 0.0003), Talose (*p* = 0.0008), etc. (Figure 7).

## 5. Discussion

In this study we investigated the gut microbiota from elderly patients undergoing orthopedic surgery. Considering confounding factors, the general information of participants including relevant previous history, laboratory test results and postoperative pain NRS were collected, and there were no differences in variables age, gender, type of surgery, blood glucose, BUN, INR, LVEF, NRS, number of hypertension, diabetes, HLP, stroke, ECG ST segment ischemia between the two groups. The variable age was divided into 4 sections with a 5-year interval. The majority is from 66 to 75 years old, and there was no difference in 4 sections between groups. Which indicated the morbidity of POCD in the elderly patients was equal in the 66–85 years interval. The patients’ gender, cardiac function, renal function, stroke history, blood glucose, lipid level and postoperative pain did not show influences in the POCD morbidity in this study, which excluded the confounding factor and provided us a focus on the role of gut microbiota.

Although alpha diversity and beta diversity of gut microbiota were similar in two groups, but at the genus level, 20 gut bacterium were significantly altered in POCD patients compared with those in control patients. And among the 20 genus, the relative abundance of the genera *Helicobacter, Alistipes, Barnesiella, Lachnospiraceae_NK4A136_group, Prevotellaceae_NK3B31_group* and *Pediococcus* were higher in POCD group than those in Control group, and all have got significantly diagnostic efficiency analyzed by the ROC curves, which might be potential target bacterium to diagnose or predict elderly POCD. The alterations above were consistent with the recent research. As is well known, *Helicobacter pylori* (*H*. *pylori*) is a Gram-negative bacterium resides in the gastrointestinal tract starting from early age. *H*. *pylori* infection might be a risk factor for cognitive decline in the elderly (Han et al., 2018; Cárdenas et al., 2019). The biological mechanisms include reduced absorption of folate and vitamin B-12 and increased homocysteine (Berrett et al., 2018). Ren et al. (2020) claimed that the abundance of genera *Barnesiella* and *Alistipes* negatively correlated with cognition ability. Tran et al. (2019) detected an association of *Prevotellaceae* and several butyrate-producing genera with Apolipoprotein E genotypes, which is the strongest genetic risk factor for Alzheimer’s disease. Nevertheless, it was reported that decreased abundance of the *Lachnospiraceae* family was associated with cognitive decline (Liu et al., 2019). These results indicated alterations of abundance of gut microbiota play an important role in the pathogenesis of POCD in elderly patients.

Metabonomics analysis was performed to clarify whether there were associations between the metabolites produced by gut microbiota and POCD in elderly. 27 differential metabolites were screened out in the GC–MS platform, including acetic acid, arachidic acid, leucine, pyrophosphate, etc. In this study, acetic acid from gut microbiota was highly enriched to pathways of protein digestion and absorption, alcoholic liver disease, glyoxylate and dicarboxylate metabolism, glycosaminoglycan biosynthesis – heparan sulfate/heparin.

Acetic acid is the smallest short-chain fatty acid (SCFA) which can form acetylCoA and participate the metabolism of carbohydrates and fats. It is produced and excreted by certain acetic acid bacteria, notably the Acetobacter genus and Clostridium acetobutylicum. Zheng et al. (2021) built the model of decreased gut acetate level by vancomycin exposure in mice, which lead to reduction of hippocampal synaptophysin level and impaired learning and memory. This alteration might be mediated by the vagus nerve stimulation. Consistent with the recent study, we found the expression level of acetic acid was lower in POCD group than that in Control group, which indicated acetic acid was positively correlated with cognition ability, probably *via* regulating hippocampal synaptophysin level in the elderly patients undergoing orthopedic surgery.

Arachidic acid is an important component of neuronal membranes, and regulate neurotransmission, neuroinflammation, cell survival and cognition function (Martin et al., 2016). The oxidative arachidic acid metabolic pathway is the core of inflammation, and the main cause of working memory impairment leading to AD pathogenesis (Chen et al., 2022). In this study, arachidic acid was highly enriched in biosynthesis of unsaturated fatty acids pathway, and expression level was lower in POCD group in the LC–MS platform. The result inferred that arachidic acid was probably metabolized into prostaglandins and leukotrienes which mediated neuroinflammation, and finally led to cognition impairment in the elderly POCD.

Pyrophosphate was highly enriched to pathways of oxidative phosphorylation, parkinson disease, neurodegeneration – multiple diseases, and expression level was higher in POCD group. Bisphosphonates interact and regulate calcium ions which play a role in neurodegenerative diseases (Zameer et al., 2018), the result was consistent with recent study.

## 6. Conclusion

Gut microbiota altered in the elderly POCD and some of the genera bacterium might be effective diagnosis indicators to predict POCD incidence before surgery. The differential metabolites including acetic acid, arachidic acid and pyrophosphate etc. played important roles in the cognition impairment in POCD patients.

The limitation of this study is that although we have screened out the differential microbiota and metabolites which might be indicators of the POCD, a verification work has not been done yet. We planned to complete the work by fecal microbiota transplantation in future to investigate the therapeutic effect of the indicators.

## Data availability statement

The datasets presented in this study can be found in online repositories. The names of the repository/repositories and accession number(s) can be found in the article/Supplementary material.

## Ethics statement

The studies involving human participants were reviewed and approved by Medical Ethics Committee of Tongji Hospital affiliated to Tongji Medical College, Huazhong University of Science and Technology. The patients/participants provided their written informed consent to participate in this study.

## Author contributions

JB designed and supervised the project and obtained funding. JB, YX, DH, and XC collected samples and performed neurological assessments. FG provided samples. JB, SL, GZ, and JT extracted data and performed statistical analysis. JB drafted the manuscript. AL and HX revised the manuscript for important intellectual content. All authors contributed to the article and approved the submitted version.

## Funding

This study was supported by National Natural Science Foundation of China (82001161) and Braun Foundation for Scientific Research in Anesthesia (BBDF-2019-011).

## Conflict of interest

The authors declare that the research was conducted in the absence of any commercial or financial relationships that could be construed as a potential conflict of interest.

## Publisher’s note

All claims expressed in this article are solely those of the authors and do not necessarily represent those of their affiliated organizations, or those of the publisher, the editors and the reviewers. Any product that may be evaluated in this article, or claim that may be made by its manufacturer, is not guaranteed or endorsed by the publisher.

## Footnotes

^1^ www.mocatest.org
